
JOURNAL INFORMATION
==============================
NLM Title Abbreviation: ScientificWorldJournal
Journal ID: ScientificWorldJournal
NLM Journal ID: 101131163
Journal ID: TSWJ

The Scientific World Journal

ISSN: 2356-6140
EISSN: 1537-744X
Publisher: Wiley

ARTICLE INFORMATION
==============================
PMCID: PMC6084155
DOI: 10.1100/tsw.2001.409
Article ID: 690670
Article version: 1
Subjects: Extended Abstract

The Effect on CGRP-Binding of Mutations to the Hydrophilic Residues within the First Transmembrane Region of Human Calcitonin Receptor-Like Receptor (CRLR)

Conner A. C. 1 *
Howitt S. G. 1
Wheatley M. 2
Smith D. M. 2
Poyner D. R. 1
Pharmaceutical Sciences Research Institute, Aston University, Birmingham, UK
Department of Biosciences, University of Birmingham, Birmingham, UK
*Corresponding author.
Publication date: 2001
Electronic publication date: 2001 Oct 17
Volume: 1
First page: 39
Last page: 39
Copyright: Copyright © 2001 A. C. Conner et al.
Copyright year: 2001
License: This is an open access article distributed under the Creative Commons Attribution License, which permits unrestricted use, distribution, and reproduction in any medium, provided the original work is properly cited.
License URL: https://creativecommons.org/licenses/by/3.0/

==============================
4th International Meeting on Calcitonin Gene-Related Peptide (CGRP)
*TheScientificWorld* (2001) 1(S1), 39
ISSN 1532-2246; DOI 10.1100/tsw.2001.409

### THE EFFECT ON CGRP-BINDING OF MUTATIONS TO THE HYDROPHILIC RESIDUES WITHIN THE FIRST TRANSMEMBRANE REGION OF HUMAN CALCITONIN RECEPTOR-LIKE RECEPTOR (CRLR)

A.C. Conner*, S.G. Howitt*, M. Wheatley**, D.M. Smith**, and D.R. Poyner*
* Pharmaceutical Sciences Research Institute, Aston University, Birmingham, U.K.;
** Department of Biosciences, University of Birmingham, Birmingham, U.K.

The molecular basis of type II GPCR-ligand interactions has been studied extensively via the use of site-directed point mutations. The mutation of hydrophilic residues within the transmembrane regions of the secretin-receptor, for example, led to a marked reduction in ligand-affinity. This approach has been used to investigate the role of hydrophilic residues within the transmembrane domains of CRLR. The four hydrophilic amino acids within the first transmembrane domain of CRLR, (H128, S131, S134, and S138) were mutated to alanine. The genes encoding the mutant and wild-type CRLRs contained within the pcDNA3- mammalian expression vector were transiently transfected into an available RAMP1-transformed HEK293 cell-line. Point mutations were created using the Stratagene Quick-change Mutagenesis method. Transient transfections were prepared according to the Clontech CalPhos Mammalian Transfection System. Transfected cells were homogenised and CGRP-affinity measured via the competition for $^{125}$I-human CGRP by unlabelled human CGRP. All four mutants within the first transmembrane domain of human CRLR displayed *in vitro* CGRP-binding affinities which were comparable to wild-type. Average $IC_{50}$ values were 3.84 nM for wild-type CRLR and 1.29, 5.96, 4.66, and 13.4 nM for the CRLR mutants, H128A, S131A, S134A, and S138A, respectively. It is likely that hydrophilic residues within the transmembrane domains of CRLR will be involved in the recognition or action of CGRP. This study suggests that individual residues such as this within the first transmembrane helix of CRLR are not crucial for CGRP-recognition or RAMP1 interaction. We are currently investigating the remaining helices.
